# The Impact of Social Capital on Socially Responsible Supply Chain Performance: The Moderating Role of Supply Chain Transparency

**DOI:** 10.3390/foods12193624

**Published:** 2023-09-29

**Authors:** Hua Liu, Guangyao He, Ruili Ma, Shaoling Fu

**Affiliations:** College of Economics & Management, South China Agricultural University, Guangzhou 510642, China

**Keywords:** social capital, supply chain transparency, socially responsible supply chain performance, income increase, social sustainable supply chains

## Abstract

The outbreak of COVID-19 has brought global poverty to the forefront, and existing research suggests that socially responsible supply chains play an important role in poverty alleviation. However, there is limited research on how to improve the performance of socially responsible supply chains. This study innovatively chooses a dual perspective, i.e., companies and farmers in contract farming, the dominant model of socially responsible supply chains in Chinese agriculture, as the research object. Furthermore, it examines the role of social capital on the performance of socially responsible supply chains, as well as the moderating role of supply chain transparency, in order to find out how to improve the stakeholder performance. The empirical results found that the factors affecting socially responsible supply chain performance differed between the dual perspectives. From the firm’s perspective, all three dimensions of social capital (shared values, communication and reciprocity) have a significant positive effect on socially responsible supply chain performance (income increase), while supply chain transparency only positively moderates between communication and income increase. From the farmers’ perspective, only reciprocity and shared values had a significant positive effect on income increase; interestingly, supply chain transparency negatively moderated the relationship between reciprocity and income increase. This study expands the role of social capital theory, and the dual perspective examination provides insights for performance improvement of companies and farmers in socially responsible supply chains, as well as guidance for promoting sustainable social development.

## 1. Introduction

Poverty is a global problem that hinders human development and is a major challenge for developing countries. Eradicating poverty in all its forms and dimensions is the main objective of the Sustainable Development Goals (SDGs) proposed by the United Nations in 2014 [1]. In particular, the outbreak of COVID-19 has brought the issue of poverty to the forefront [2]. The UN SDG report for 2022 shows that the COVID-19 pandemic has led to the reversal of more than four years of progress in poverty eradication, with 93 million more people falling into extreme poverty in 2020. It is therefore important to know how to ensure the dual integration of poverty reduction and sustainable development. The majority of the world’s poor (about 80%) live in rural areas and depend mainly on agriculture for their livelihoods [3]. Although China has achieved the task of eradicating absolute poverty, poor farmers generally lack the capacity to survive, some of the farmers who have escaped poverty are at risk of returning to poverty, and the sustainable stability of farmers’ escape from poverty faces certain challenges [4].

In recent years, sustainable supply chains have received much attention from scholars and practitioners [5,6]. Some scholars have pointed out that socially responsible supply chains play an important role in alleviating poverty among farmers [7,8]. By including relatively poor farmers in the supply chain as producers or distributors, leading companies can create economic value for farmers and themselves in socially responsible supply chains [7], thereby achieving stability while addressing poverty. In China, contract farming (C+F) is a typical socially responsible supply chain model for agribusinesses to support poor farmers. Contract farming (C+F) has been defined as “a system of producing and supplying agricultural products under a forward contract, the essence of which is a commitment to provide a certain quantity of a certain type of agricultural product at a time and price that is known to be required by the purchaser” [9]. However, Dan et al. [10] found that, in reality, Chinese farmers involved in socially responsible supply chains experienced only a modest 2.77% increase in income. However, there is a lack of research on how to improve the performance of socially responsible supply chains, so it is important to explore the mechanisms at play to promote the socially responsible supply chain performance in developing countries, and this study attempts to fill this research gap in order to address poverty reduction and sustainable development goals, and to provide empirical evidence in this regard.

Social capital is often regarded as an effective guarantee for firms to improve their performance [11], because firms with high-quality social capital can improve the efficiency of cooperation to reduce risks by establishing close ties or reaching consensus with cooperation subjects [12]. Meanwhile, the essence of social capital is valuable resources accumulated through frequent inter-organisational interactions [13]. These resources are specific, well-defined and dynamic, resulting in stable, long-term and mutually supportive cooperation [14]. Liu et al. [15] found that in pro-poor industrial, farmers’ social capital can enhance their livelihood capital. Moreover, the returns to social capital exceed those to physical or human capital [16]. However, social capital may play different roles in different types of business relationships [17]. While we do not know whether social capital can improve the performance of socially responsible agricultural supply chains, this research gap has an important role to play in realising the sustainable development of socially responsible agricultural supply chains in the context of developing countries.

Transparency has gained renewed attention in the current era of rapidly advancing digital technologies [18,19]. Efficient supply chain transparency is a crucial factor in maintaining or regaining the trust of supply chain partners [20]. At the same time, supply chain transparency is a key driver of efficient supply chains and can fulfil the potential of removing information barriers in cooperative relationships. Therefore, this study selects companies and farmers in the “C+F” model and empirically examines the role of social capital on the performance of socially responsible supply chains using structural equation modelling, as well as the moderating role of supply chain transparency, in order to discover ways to achieve increased incomes for both companies and farmers. This study, in an attempt to address the problem of poverty in developing countries, seeks to reveal the factors affecting the performance of socially responsible supply chains to inform the achievement of poverty reduction and sustainable development. This study expands the scope of application of social capital theory and reveals the similarities and differences in the roles of social capital on both sides of the supply chain co-operation through a dual perspective, which makes up for the previous research on unilateral perspectives of social capital and also provides inspiration for subsequent research on the differences in the roles of social capital. The findings inform how agribusinesses and farmers can use social capital to enhance socially responsible supply chain performance, and how social capital can be used appropriately in the context of supply chain transparency. This study explores the following two questions:

(1) What is the role of the three dimensions of social capital (shared values, communication and reciprocity) on socially responsible supply chain performance (income increase)?
(2) How does supply chain transparency moderate the relationship between the three dimensions of social capital (shared values, communication and reciprocity) and socially responsible supply chain performance (income increase)?

The remainder of this paper is organised as follows: Section 2 presents a comprehensive literature review and outlines the research hypotheses. Section 3 describes the methodology. Section 4 presents the analysis and results. Section 5 provides a discussion of the findings and their implications. Section 6 concludes the paper with conclusions and limitations.

## 2. Literature Review and Research Hypotheses

### 2.1. Social Capital

Social capital refers to “the sum of the actual and potential resources embedded within, available through, and derived from the network of relationships possessed by an individual or social unit” [21]. Initially, social capital was studied at the individual level to describe the accumulation of relational resources through personal interactions [22]. Subsequent research shifted the focus towards social capital being embedded in supply and demand relationships [23,24], and supply chain relationships [25,26,27,28]. The main focus in supply chain relationships is on the strengths of social capital, including improvements in innovation capabilities [29], performance outcomes [14,30], risk mitigation [31], and organizational resilience [13]. Table 1 shows a selection of studies on the impact of social capital on performance.

Although the undisputed importance of social capital in supply chains, we have identified three critical research gaps that warrant further investigation. Firstly, there are few studies on the role of social capital in the context of “C+F”. In this supply chain context, companies and farmers engage in collaborative resource pooling for joint production, aimed at elevating the livelihoods of vulnerable farmers, concurrently achieving the company’s social responsibility and economic performance objectives [32]. Social capital theory provides a theoretical perspective on how companies and farmers use each other’s social networks to increase their income.

Secondly, the majority of existing studies have only examined social capital embedded in unilateral relationships [13,33,34,35]. Buyers and suppliers have different views on the cooperative relationship [36], and this divergence is even more pronounced in the “C+F” context [12]. The significant differences between farmers and companies in terms of power, resources, and capabilities make it necessary to explore the role of social capital both from the perspective of companies and the perspective of farmers. In this study, the relationship between social capital and performance was empirically investigated from both companies and farmers’ perspectives.

Thirdly, fewer existing studies have focused on different dimensions of social capital simultaneously. Nahapiet and Ghoshal divide social capital into three dimensions: cognitive, structural and relational [21]. The cognitive dimension includes shared values, interpretations, common language, shared codes and systems of meaning between parties. The structural dimension reflects the overall pattern of interaction of the network structure [37], for example a supply chain can be seen as a network structure that connects customers and suppliers. The relational dimension is developed through previous interactions and further generates trust, obligation and reciprocity. However, the majority of existing research focuses on the relationship dimension and ignores the other two dimensions [28]. This study simultaneously explores the role of the three dimensions of social capital on socially responsible supply chain performance. Firstly, shared values are used to measure the cognitive dimension, as suggested by Lin and Lu [38] that shared values are the key dimension of cognitive social capital. Shared values facilitate mutual understanding of goals and norms between supply chain partners [39]. However, the farmers in this study lack formal organization, making it difficult to establish shared interpretation, language and system of codes and meanings. Some studies have found that shared values contribute to reducing monitoring of suppliers and thus benefit performance [34]. Secondly, structural capital is thought to improve communication between supply chain partners and facilitate a better understanding of each other’s key processes and operations [40]. In contrast, poor communication can lead to conflict and misunderstanding, which often hinder cooperative efforts. Communication serves as the glue that holds supply chain partners together [41], therefore, this study focuses on communication in structural capital. Thirdly, reciprocity facilitates the maintenance of supply chain relationships. However, in the past, relational capital has mainly examined the trust dimension, with little focus on the important role of reciprocity. Given this the specific cultural context of human relations in China and the reciprocal use of resources between companies and farmers, which makes the occurrence of reciprocity is inevitable, we have chosen reciprocity as a measure of relational capital.

**Table 1:** A summary of the impact of social capital on performance.

Context	Perspective	Dimensions of Social Capital	Findings	Reference
Manufacture	213 manufacturers	Relational	Both supplier relationship capital and customer relationship capital can affect a manufacturer’s operational.	[33]
Manufacture	203 manufacturers	Relational, structural and cognitive	Different impacts of supply chain social capital on performance in the supplier and the customer sides.	[14]
Manufacture	117 industrial companies	Relational	Dependence moderates the relationship between social capital and operating performance.	[30]
Manufacture	206 manufacturers	Relational, structural and cognitive	Relational and structural capital accumulation have a positive effect on economic performance, while cognitive capital accumulation has no significant effect on economic performance.	[35]
Manufacture	308 manufacturers	Relational	Relationship capital has a positive impact on suppliers’ operational performance.	[24]
Retailing	393 distributors	Relational, structural and cognitive	All three dimensions of social capital can positively influence buyer performance, while business and political ties can moderate these relationships.	[42]
Manufacture	276 manufacturers	Undimensioned	In traditional manufacturing firms, social capital indirectly enhances operational performance through knowledge acquisition.	[43]
Retailing	12 retailers and 70 suppliers	Relational, structural and cognitive	The impact of each dimension of social capital on the operational performance of retailers and suppliers is significantly different, and the impact of the same dimension is different for retailers and for suppliers.	[40]

### 2.2. Supply Chain Transparency

Although many studies have verified that social capital positively affects supply chain performance [14,30,34], this does not always seem to hold true. Some studies suggest that social capital may have a negative impact on supply chain outcomes [44,45]. Therefore, there may be moderating variables affecting the role of social capital. In this study, transparency, an important influencing factor in socially responsible supply chains, was selected as a moderating variable to test its effect on the relationship between social capital and socially responsible supply chain performance.

Supply chain transparency refers to a company’s disclosure of information to the public (including consumers and investors) about its upstream operations and the products it sells. Its role is to potentially change the supply chain and how it is managed, reducing risk and increasing efficiency [46]. Supply chain transparency can be categorised into three types: historical transparency, strategic transparency and operational transparency [47]. Operational transparency refers to a company’s disclosure of its operational processes [48]. Specifically, operational transparency entails disclosing the “behind-the-scenes” work that a company carries out through its operational processes [49]. In “C+F”, information sharing between the company and the farmer primarily occurs at the operational level. Therefore, this study adopts operational transparency as the measure of supply chain transparency, focusing on the exchange of sales information, demand forecasts, inventory information, and production planning information between the company and the farmer.

### 2.3. The Impact of Different Dimensions of Social Capital on Socially Responsible Supply Chain Performance

Socially responsible supply chain performance is measured by income increase resulting from increased competitiveness, including increased production capacity, increased cooperative selling power and increased return on investment.

#### 2.3.1. The Impact of Shared Values on Income Increase

Shared values are developed through a continuous and self-reinforcing process of participatory meaning-making, as all parties construct a common understanding [50]. When shared values exist between firms and suppliers, their understanding of interests and goals are more aligned [26,51]. In this case, the motivation to collaborate between the firm and the supplier is increased, while the likelihood of conflict and risk is reduced [45], resulting in higher revenue levels. When both partners in the supply chain have similar management styles and cultural backgrounds, they are more likely to pursue collective gains, thereby discouraging harmful actions. Conversely, when goals and values are misaligned, interactions between the parties can lead to misunderstandings and conflicts over events [51]. As misunderstandings and conflicts escalate, both parties become dissatisfied with the relationship, limiting information sharing and consequently negatively affecting productivity and performance [25]. Therefore, in socially responsible supply chains where shared values are present, the company and the farmer will agree on what constitutes an improvement in income enhancement, the approaches to achieving improvement, and the potential barriers to achieving performance, thereby contributing to income enhancement. On this basis, it is hypothesised that:

**H1a.**  *Shared values are positively related to income increase in the company–farmer relationship.*

#### 2.3.2. The Impact of Reciprocity on Income Increase

Wu et al. [52] argue that the concept of reciprocity is a potential mechanism for exchange whereby when one person gives some resources to another, an obligation is created for the latter to return resources of comparable value to the former at some point in the future. Previous research emphasises that social reciprocity helps to establish, develop and maintain successful relational exchanges [53]. However, the absence of reciprocity between partners poses a risk to collaboration and reduces its stickiness. Uncertainty in such relationships will lead members to withhold resources, making further collaboration more difficult. In reality, reciprocity between companies and farmers manifests itself mainly in the following ways: when there is a significant difference between the market price and the contract price, both parties make appropriate concessions, thereby reducing the losses of both partners. In the event of natural disasters or other irresistible and unexpected risks, the company and the farmer are willing to share responsibility and support each other in times of difficulty. Although such reciprocal behaviour may be detrimental to maximising their individual interests, it increases their willingness to cooperate and mitigate the various costs and unnecessary losses resulting from a short-term focus on self-interest, and ultimately promotes increased income. Therefore, we propose the following hypothesis:

**H1b.**  *Reciprocity is positively related to income increase in the company–farmer relationship.*

#### 2.3.3. The Impact of Communication on Income Increase

Communication refers to the process of contact and message transmission between supply chain partners, including aspects such as frequency, direction, mode and influence strategy [41]. Sharing information can reduce the bullwhip effect in supply chains [54]. Effective communication can help to enrich one’s own knowledge resources by broadening access to information and knowledge from partner firms, and having this information is valuable in a competitive environment, e.g., derived from key information such as orders, demand, cost and quality. Furthermore, communication is important for managing supply risks, as buyers need access to key sources of information to assess the impact of risks in advance, so that they can take immediate action to improve decision making, order accuracy, facilitate effective coordination of processes, reduce inventory turbulence and contribute to supply chain performance [45]. In the “C+F” model of socially responsible supply chains, communication with farmers allows companies to keep abreast of farmers’ production, so that they can make production adjustments and provide technical advice to farmers. This facilitates the timely identification and resolution of production problems, ensuring the quality of agricultural products and improving incomes for both parties. The farmers’ initiative in communicating with the company during production will not only improve production results, but will also strengthen cooperation between the two parties, thereby reducing production risks and increasing income. Therefore, the hypothesis was formulated that:

**H1c.**  *Communication is positively related to income increase in the company–farmer relationship.*

### 2.4. The Moderating Effect of Supply Chain Transparency on the Relationship between Social Capital and Income Increase

This study proposes that supply chain transparency positively moderates the relationship between social capital and income increase. In instances where there is high supply chain transparency between a company and a farmer, the company gains a clear understanding of the farmers’ inputs, the use of production materials and whether the company’s requirements are strictly followed. A high level of transparency provides direct insight into the production behaviour of farmers, and similar production practices encourage the development of shared values between the company and the farmer. As a result, higher levels of transparency contribute to income increase derived from shared values. Moreover, transparent farmer production practices strengthen the impact of reciprocity on income increase. As supply chains become more transparent, the value of reciprocity becomes more pronounced, leading to greater income gains. With a higher degree of supply chain transparency between the company and the farmer, the company and the farmer have access to more information about production, marketing, and other relevant aspects. This enhanced information exchange promotes trust between the parties and improves the efficiency of communication, which ultimately influences income outcomes. This is also true from the farmers’ perspective. In summary, when supply chain transparency is high, the effect of the three dimensions of social capital on income increases is amplified. Therefore, the following hypothesis is proposed:

**H2a.**  *Supply chain transparency positively moderates shared values and income increase in the company–farmer relationship.*

**H2b.**  *Supply chain transparency positively moderates reciprocity and income increase in the company–farmer relationship.*

**H2c.**  *Supply chain transparency positively moderates communication and income increase in the company–farmer relationship.*

**Figure 1:**
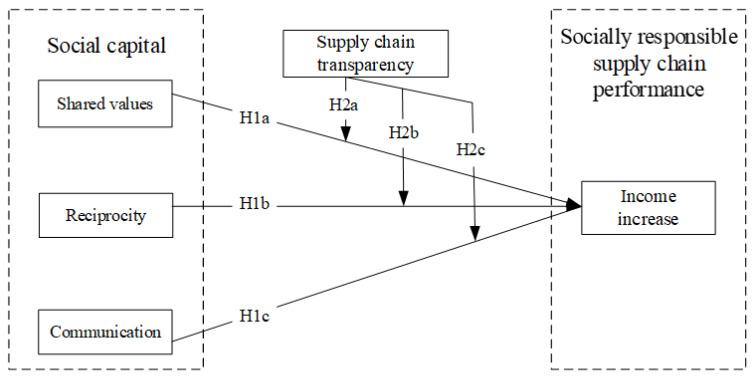
Theoretical model.

In summary, this theoretical model is shown in Figure 1.

## 3. Materials and Methods

### 3.1. Methods

In order to analyse the relationship between social capital and socially responsible supply chain performance, partial least squares-structural equation modeling is applied. PLS-SEM is a causal predictive SEM method designed to provide causal explanations. PLS-SEM can estimate models with complex structures and does not require imposing distributional assumptions on the data [55,56]. Compared with covariance-based structural equation modeling (CB-SEM), PLS-SEM has higher statistical power and is easier to achieve convergence [57]. In addition, if the size of the dataset is limited, PLS-SEM shows higher robustness than CB-SEM when the data are non-normal [58].

Evaluating PLS-SEM results involves a two-step approach: (1) assessing the outer (measurement) models, and (2) examination of the inner model (structural relations among the latent factors) [59]. The former is used to estimate associations between latent variables and observed indicators, while the latter is used to test causality between latent variables.

### 3.2. Sampling and Data Collection

This study used a questionnaire research method to validate the conceptual model. The questionnaire consisted of two parts: the basic profile of the respondents and the measurement items. The measurement items were adapted from well-established scales used by domestic and international scholars, and were compared with each other in English and Chinese to correct for differences.

Data collection for this study was a two-stage process that took a total of four months. In the first stage, data on agribusinesses were collected using random sampling. In the second stage, a modified snowballing technique was used to collect data from offline farmers working with the agribusinesses. Excluding invalid questionnaires, a total of 201 valid questionnaires were obtained from the company data, with a valid questionnaire return rate of 71.7%, and 461 valid questionnaires were obtained from the farmers’ data, with a valid questionnaire return rate of 92.2%. The sample size met the criteria for conducting empirical research.

As with all self-reported data, there is a potential for common method biases [60]. Harman’s single-factor test was used to examine the possibility of common method bias. The results showed the presence of several factors with eigenvalues greater than 1.0, and the largest variance accounting for less than 50% of the variance (companies’ perspective: 47.941%; farmers’ perspective: 35.668%). Thus, the common method bias is not a serious problem in our data [61].

Table 2 shows that the type of operation of the enterprises in this survey is mainly private enterprises, accounting for 59.2%; the time of cooperation with farmers is mainly less than 10 years, accounting for 77.1%; the number of cooperative farmers is less than 1000, accounting for 61.7%; the number of agricultural products purchased each year is 11 times or more, accounting for 22.9%; 34.3% of the enterprises believe that the trust between the two sides has reached a certain level.

From Table 3 it can be seen that the level of education of the farmers is mainly concentrated in junior high school with 45.6%. Only 2.2% were educated above high school, which shows that most of the farmers involved in agricultural production have a low level of education. The sample shows that cooperation between farmers and companies is still at an early stage. Overall, 43% of the farmers said that they had reached a certain level of mutual trust, which gives farmers and companies a sense of a bright future and gives confidence to researchers studying company–farmer cooperation.

### 3.3. Measures

The variables were measured on a 7-point Likert scale using three or more measure items for each variable. There are five structural variables in the questionnaire, with the three dimensions of social capital referenced to measures by Nahapiet and Ghoshal [21], Villena et al. [45], Zhang et al. [35], Wu and Chiu [27], Zhang et al. [43]; supply chain transparency referenced to Narasimhan and Kim [62], Stanley and Wisner [63]; income increases refer to Lai et al. [64], Zhang et al. [35]. The scale measures and sources are shown in Table 4.

## 4. Analysis and Results

### 4.1. Reliability and Validity Analysis

This study used SPSS25.0 and SmartPLS3.0 software to test the reliability and validity. Cronbach’s α values were selected to test the internal consistency, as shown in Table 5, except for the shared values of the company’s perspective which were slightly below 0.6, the Cronbach’s α values of all other variables were greater than 0.6; and the CR values were greater than 0.7. The results of the validity analysis are shown in Table 6 and Table 7. In these tables above, the factor loading values for all variables were greater than 0.5 and the t-values were greater than 2, reaching a significant level; the mean extracted variance values for all factors were greater than 0.5, indicating that the aggregated validity of the model was good. In Table 8 and Table 9, the correlation coefficient values between each variable and the other variables are all less than the square root of the AVE value for that variable, indicating good discriminant validity.

### 4.2. Structural Equation Modelling and Results

**Figure 2:**
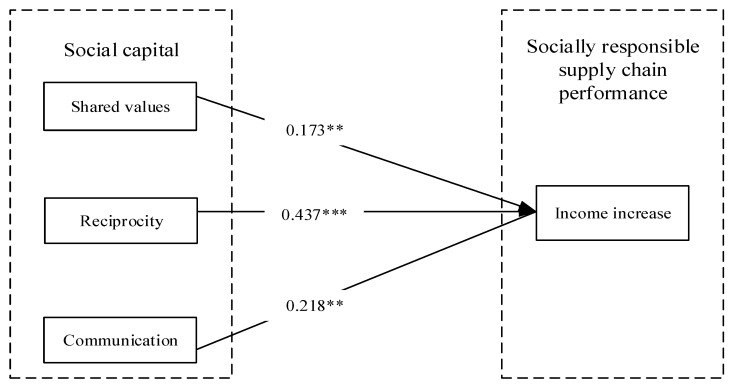
The standard path coefficient of the model: company’s perspective. Note. ** *p* < 0.01, *** *p* < 0.001.

**Figure 3:**
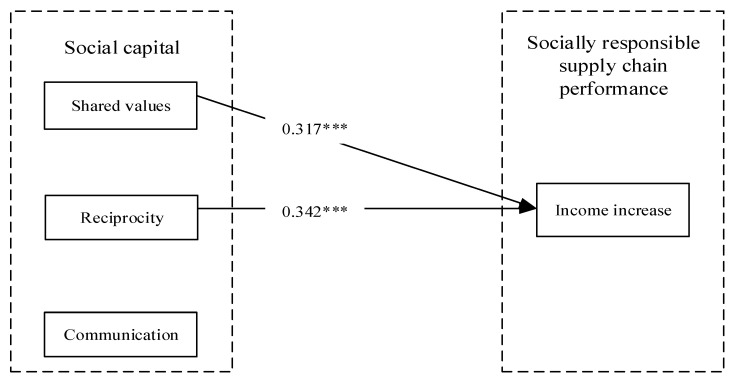
The standard path coefficient of the model: farmers’ perspective. Note. *** *p* < 0.001.

SmartPLS 3.0 statistical analysis software was used to test the relationship between social capital, supply chain transparency, and income increase in the company’s and farmers’ perspectives. The standard path coefficients of the structural equation model are shown in Figure 2 and Figure 3. H1a, H1b, H1c and H2c were supported for the company’s perspective in this study, whereas only H1a and H1b were supported for the farmers’ perspective.

The empirical results show that the three dimensions of social capital (shared values, reciprocity, and communication) have different effects on socially responsible supply chain performance (income increase). From the company’s perspective, shared values (β = 0.173, *p* < 0.01), reciprocity (β = 0.437, *p* < 0.001) and communication (β = 0.218, *p* < 0.01) all have a significant positive effect on income growth and H1a, H1b and H1c are supported, whereas from the farmers’ perspective, only shared values (β = 0.317, *p*< 0.001) and reciprocity (β = 0.342, *p* < 0.001) had a significant positive effect on income increase, H1a and H1b were supported, while H1c was not supported.

### 4.3. Moderating Effect of Supply Chain Transparency Analysis

**Figure 4:**
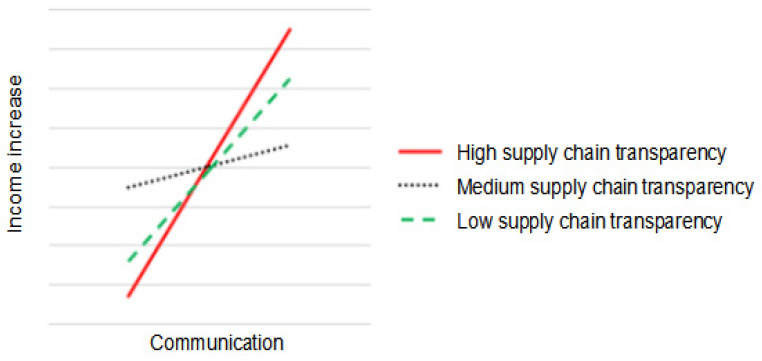
The moderating effect of supply chain transparency on the relationship between communication and income increase: company’s perspective.

**Figure 5:**
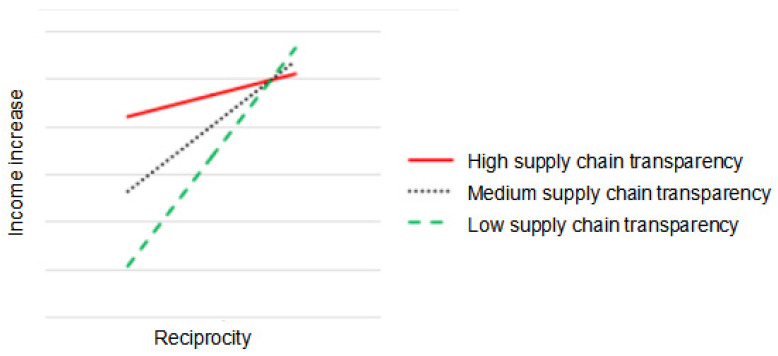
The moderating effect of supply chain transparency on the relationship between reciprocity and income increase: farmers’ perspective.

The moderating effects of supply chain transparency are shown in Table 10, Figure 4 and Figure 5. From the company’s perspective, high levels of supply chain transparency are more conducive to the impact of communication on income increase than low levels of supply chain transparency. From the farmers’ perspective, low levels of supply chain transparency are more conducive to the impact of reciprocity on income increase than high levels of supply chain transparency.

This study finds that the moderating role of supply chain transparency between social capital and “C+F” income increase is different in the dual perspective. From the company’s perspective, supply chain transparency does not have a moderating role between shared values, reciprocity and income increase, and H2a and H2b are not supported. However, there is a significant positive moderating effect of supply chain transparency between communication and income increase, which is consistent with the hypothesis of this study that supports H2c. The empirical results illustrate that a high level of supply chain transparency is conducive to promoting income increase when there is communication between the company and the farmer. From the farmers’ perspective, the moderating effect of supply chain transparency in the relationship between shared values and communication on income increase is not significant, and H2a and H2c are not supported. There is a significant negative moderating effect in the relationship between reciprocity and income increase, and H2b is not supported. The empirical results suggest that when farmers and companies establish reciprocal relationships, low levels of supply chain transparency are conducive to promoting income growth between them.

## 5. Discussion and Implications

### 5.1. Improving Socially Responsible Supply Chain Performance through Social Capital

The impact of different dimensions of social capital on socially responsible supply chain performance (income increase) differs depending on the perspective. From the firm’s perspective, the ranking of the impact on income increase is as follows: reciprocity, communication, shared values. This result is consistent with the study by Cai et al. [65], who found a positive relationship between social capital and fundraising performance. Conversely, from the farmers’ perspective, only communication had no significant effect on income increase. This result is consistent with the findings of Jääskeläinen et al. [17] that structural capital does not always directly benefit supplier performance management. In addition, reciprocity emerges as the most influential dimension on income increase from both perspectives, probably due to China’s status as a developing country where rapid economic development remains a primary goal. Profit maximisation is the main objective for companies, while many farmers are relatively impoverished, making reciprocity crucial for the survival and growth of both parties. Furthermore, as China is a collectivist culture that focuses on relational interaction, reciprocity is a tangible expression of the relationship. Therefore, when both parties achieve a certain level of reciprocity, their cooperation is strengthened, leading to increased income. Reciprocity is also an expression of social responsibility, as companies support farmers and create a win-win situation for both parties. In practice, some companies, such as the Guangdong WENS Group, use reciprocity to work with farmers, especially during natural disasters. In such cases, the company is willing to bear the main losses and takes active measures to help farmers overcome difficulties. If the market price is higher than the contract price, the company will increase the contract price accordingly and take additional measures to protect farmers’ income. As a result, farmers will be more willing to cooperate in production, thereby securing the income of both parties. Therefore, it is recommended that both parties give priority to cultivating reciprocal behaviour in their cooperation, looking beyond short-term gains and focusing on long-term goals of increasing incomes.

Interestingly, we observe that the factors with the second highest degree of influence on income increase differ in the dual perspective. From the company’s perspective, communication plays a secondary role, while the farmers’ perspective is shared values. This result is consistent with the findings of Fu et al. [12]. The possible reason behind this divergence could be attributed to “C+F”, where the company, as the buyer, plays a dominant role in communication and is able to proactively communicate various information to the farmer in a timely manner. This plays a key role in production and thus influences the final output. Possible reasons for the extent to which shared values play a role in the farmers’ perspective are that farmers have traditional empirical knowledge of agricultural production, and they find it difficult to accept different new technologies and ideas. Therefore, it is easier to collaborate with companies that share the same values and production philosophy, which helps avoid misunderstandings and conflicts.

In contrast to the results of previous studies [12], communication does not have a significant impact on performance from the farmers’ perspective. We note that this may be due to the fact that the farmer, as a supplier, has a weaker position compared to the dominant role of the company in the “C+F” model. As a result, it is difficult for the farmers’ communication to receive sufficient attention from the company. In addition, the “one-to-many” relationship implies that companies may face information overload if they have to receive feedback from many farmers, which may affect their efficiency. The sample statistics show that almost half of the farmers have only a secondary education, which may contribute to inefficient communication. The combination of these results in unsatisfactory communication from the farmers’ perspective.

### 5.2. The Moderating Effect of Supply Chain Transparency

From the company’s perspective, supply chain transparency does not have a moderating effect on the relationship between shared values, reciprocity and income increase. This may be due to the fact that when the company has a high level of supply chain transparency with farmers, the farmers’ transparent production behaviour must also be pre-approved by the company. Consequently, the influence of shared values is weakened in such cases, as our measure of shared values implies that the company identifies with the farmers’ production practices. Similarly, transparency of farmers’ production behaviour will also reduce the role of reciprocity in increasing income, because when all behavioural systems are transparent, there is less room for manipulation and farmers will reduce the use of reciprocity. Even if farmers engage in reciprocity, companies cannot reward individual farmers with high levels of supply chain transparency. Thus, when supply chain transparency is high, the effectiveness of reciprocity in increasing income is reduced.

From the farmers’ perspective, supply chain transparency has a negative moderating effect on the relationship between reciprocity and income increase. This is in line with the findings of Peschel and Aschemann-Witzel [66], who found a negative effect of transparency. The value of reciprocity is better perceived when both companies and farmers have higher levels of supply chain transparency. Excessive levels of reciprocity tie farmers into partnerships with companies, which may lead to missed opportunities for more favourable cooperation in a market economy. Higher opportunity costs undermine farmers’ individual interests and thus reduce income increase. This finding explains the argument of Villena et al. [45] that an excessive amount of social capital can be detrimental to performance. Furthermore, when both parties have transparency in the supply chain, the disclosure of information about production, marketing and management reduces the incentive for farmers to rely on reciprocity to reap rewards. Instead, they have more to lose than to gain, which can affect their incomes. In addition, Chinese farmers are poorly educated and relatively economically disadvantaged, and when the results of reciprocity are made transparent, if farmers perceive unfairness, those with limited knowledge and resources may act on short-term interests to undermine cooperation, thereby inhibiting income growth.

From the farmers’ perspective, supply chain transparency did not have a moderating effect on the relationship between shared values, communication and income increase. The possible reason for this is that when farmers and companies have supply chain transparency, farmers gain access to the company’s information about production and marketing. Both parties can better understand the role of shared values in the process of acquiring information. As mentioned above, excessive social capital is not conducive to improved performance [45]. Another reason may be that supply chain transparency allows farmers to have a clear understanding of the company’s production and sales practices, thus weakening the role of shared values. Transparent production and sales practices by the company will also reduce the role of communication, as establishing supply chain transparency requires frequent use of communication in the initial stages, which inevitably costs a lot of human and financial resources to ensure it is effective, and is thus counterproductive to increasing income. It is also possible that the frequent use of communication in establishing supply chain transparency has weakened the role of communication in social capital. Furthermore, when companies make relevant information transparent, the need for communication diminishes. Even if farmers communicate with the company, when supply chain transparency is high, important information is revealed and the role of communication is reduced for the remaining information. Therefore, when supply chain transparency is high, the impact of communication on income increase is reduced.

The observed differences in the moderating effect may be due to the unequal relationships and disparities in resources within the “C+F” model.

### 5.3. Theoretical Implications

This study makes several theoretical contributions. First, the impact of social capital on the performance of socially responsible supply chains is examined by incorporating the three dimensions of social capital into one research model. The results of the differences in the roles of the different dimensions contribute to a better understanding of the similarities and differences in the effects of the dimensions of social capital on performance, thus enriching the theory of social capital. Adopting a dual perspective to study the role of social capital in socially responsible supply chains is conducive to the expansion of social capital theory research in the supply chain field. Under the effect of the moderating variable supply chain transparency, social capital from the farmers’ perspective shows a negative effect on performance, a finding that complements existing research on the negative role of social capital.

Second, it empirically examines the moderating role of supply chain transparency, providing valuable insights into the intrinsic link between social capital and performance. By exploring this relationship, it extends the existing literature on supply chain transparency.

Third, the alliance between companies and farmers is explained in detail from a binary perspective in the context of socially responsible supply chains. This comprehensive analysis enriches the study of socially responsible supply chains and offers new research perspectives for scholars in the field.

Finally, this study fills a research gap by focusing on socially responsible supply chains in developing countries and can help supply chain management research understand how to use the framework to assess the social aspects of sustainability.

### 5.4. Managerial Implications

The empirical results have positive management implications and policy implications for enhancing income growth between “C+F” and promoting the sustainable development of socially responsible supply chains.

The management implications are as follows: First, in order to increase revenues, companies should focus on cultivating social capital with farmers. When resources are limited, developing reciprocity with farmers can be a priority, followed by communication and shared values. Companies can demonstrate reciprocity to farmers by offering profits, subsidies, dividends and support in the event of major losses or by assuming a significant proportion of the risks. Effective communication with farmers can be ensured through regular visits by technical and managerial staff, establishing various communication channels to understand farmers’ concerns, and involving them in social activities to promote shared values. Second, agribusiness managers should recognise the positive moderating effect of supply chain transparency on the relationship between communication and income growth. Therefore, improving communication with farmers where there is a high level of supply chain transparency is likely to result in a significant increase in income.

The implications for farmers are as follows: First, farmers can increase their income by developing social capital with companies. Priority should be given to developing reciprocity, followed by shared values. In times of loss, farmers can actively participate in risk sharing with the company to foster reciprocity. It is advisable for farmers to understand the company’s culture and production philosophy, and to combine the company’s philosophy with their own production experience. Additionally, to achieve a win-win state of coexistence, farmers should share their own production experience with company stakeholders to build shared values. Second, supply chain transparency has a negative moderating effect on the relationship between reciprocity and income growth. Farmers are advised to be cautious about applying reciprocity in an environment of high supply chain transparency, which would be detrimental to their own income growth.

From a government perspective, it is recommended to introduce “C+F” relationship governance as an indicator for evaluating leading companies. Evaluation criteria such as reciprocity and communication can be included to guide companies in actively fostering social capital with farmers and increasing income for both parties. In addition, the government can provide incentives for companies to establish socially responsible supply chains and encourage innovative business models that motivate farmers to participate in the supply chain.

## 6. Conclusions and Limitations

In this study, a typical model of socially responsible supply chain for agriculture in China, “C+F”, was selected for investigation. Our investigation involved collecting data from agricultural companies and farmers to empirically explore the relationship between the three dimensions of social capital (shared values, reciprocity and communication) and socially responsible supply chain performance (income increase), as well as the moderating role of supply chain transparency. Findings indicate that the impact of the three dimensions of social capital on income increase varies. From the company’s perspective, shared values, communication, and reciprocity positively and significantly influenced income increase, with reciprocity playing the most significant role, followed by communication and shared values. From the farmers’ perspective, reciprocity and shared values had a significant positive effect on income increase, whereas communication did not show a significant effect, the degree of their role is in the following order: reciprocity, shared values. Most previous studies have examined social capital embedded in unilateral supply chain relationships [13], and since buyers and suppliers have very different views of inter-organisational partnerships [14], we adopt a dual perspective. The results of the dual perspective are more conducive for companies to give targeted cultivation to the supply side social capital, thus enhancing the performance of both sides of the socially responsible supply chain. The results of this study found that the role of social capital differs between the company and farmer sides, a finding that compensates for previous examinations of the role of social capital from a single perspective and provides evidence for the result that the role of social capital is not significant.

Regarding the moderating effect of supply chain transparency, the findings suggest that different perspectives produce different moderating effects. From the firm’s perspective, supply chain transparency only positively moderates the relationship between communication and income increase. On the contrary, from the farmers’ perspective, supply chain transparency has a significant negative moderating effect on the relationship between reciprocity and income increase. This is an interesting finding. While a large number of studies have confirmed the positive effects of supply chain transparency [20], there are also studies that have found negative effects of higher social information transparency [67], and this study examines operational transparency in supply chain transparency, and the negative moderating effect of supply chain transparency from the farmers’ perspective reaffirms this conclusion.

This study contributes to the literature on socially responsible supply chain management and provides practical guidance for companies, farmers and governments. However, this study still has some limitations. Firstly, this study only examined the role of social capital on socially responsible supply chain performance, but there should be other influencing factors, and the role of the use of new technologies on performance could be considered in the future. Second, the moderating role of supply chain transparency between social capital and socially responsible supply chain performance is limited in this study. In the future, the potential mechanism of the effect of social capital on performance can be explained from other perspectives, such as the introduction of environmental uncertainty as a moderating variable. Third, socially responsible supply chain performance is only measured by income increase, which can be measured by operational performance, strategic performance, etc. in the future. Finally, this study selected the “C+F” model as the object of research on socially responsible supply chains, but there are many forms of socially responsible supply chain models in developing countries, and the performance enhancement programme of cooperatives and farmers can be considered in the future.

## Figures and Tables

**Table 2:** Basic characteristics of companies.

Variable	Variable Value	N = 201	
		Frequency	Percentage
Distribution	Guangdong province	123	61.2
	Hainan province	78	38.8
	Missing values	0	0
Number of cooperative farmers	≤50	26	12.9
	51–100	36	17.9
	101–500	56	27.9
	501–1000	6	3.0
	≥1001	74	36.8
	Missing values	3	1.5
Cooperation time (years)	≤1	12	6.0
	2–3	45	22.4
	4–5	30	14.9
	6–10	68	33.8
	≥11	40	19.9
	Missing values	6	3.0
Number of agricultural purchases	1–2	49	24.4
	3–5	35	17.4
	6–10	5	2.5
	≥11	46	22.9
	Missing values	66	32.8
Ownership	Private	119	59.2
	Joint venture	14	7.0
	Collective	7	3.5
	State owned	3	1.5
	Others	51	25.4
	Missing values	7	3.5
Cooperation stage	Collaborative performance is in a period of fluctuation	66	32.8
	Some level of trust developed	69	34.3
	A long-term relationship is established	64	31.8
	Dissatisfaction with the partnership begins	2	1.0
	Ended or in the process of ending the relationship	0	0
	Missing values	0	0

**Table 3:** Basic characteristics of farmers.

Variable	Variable Value	N = 461	
		Frequency	Percentage
Distribution	Guangdong province	140	30.4
	Hainan province	321	69.6
	Missing values	0	0
Age	≤30	41	8.9
	31–40	101	21.9
	41–50	154	33.4
	≥51	114	24.7
	Missing values	51	11.1
Educational level	Below primary	10	2.2
	Primary	71	15.4
	Junior high school	210	45.6
	High school	81	17.6
	High school and above	10	2.2
	Missing values	79	17.1
Cooperation time (years)	≤1	86	18.7
	2–3	89	19.3
	4–5	80	17.4
	≥6	85	18.4
	Missing values	121	26.2
Cooperation stage	Collaborative performance is less consistent	55	11.9
	Trust has reached a certain level	198	43.0
	A long-term relationship is established	92	20.0
	Dissatisfaction with the collaboration begins	9	2.0
	Ended or in the process of ending	17	3.7
	Missing values	90	19.5

**Table 4:** Measurement items of companies and farmers.

Constructs	Meaures		Sources
	Company’s Perspective	Farmers’ Perspective	
Shared Values	The company identifies with the production methods used by the farmer	I identify with the management practices used by the company	[21,27,35,43,45]
	The company does what the farmer wants because it agrees with the farmers’ production practices	I follow what the company wants to do because I have similar ideas to theirs about how to manage the company	
	The company feels that the farmer sees the company as an “important member of their team” and not just a buyer	I feel that the company sees us as “important members of their team” and not just as producers	
Reciprocity	The farmer is most helpful to the company when circumstances change	The company gives us maximum assistance when things change	
	Collaboration reduces the need for fixed assets	Working with each other reduces the investment in fixed assets	
	Cooperation reduces capital investment	Working with each other reduces capital investment	
	The company can often receive good advice from the farmer	I can usually receive good advice from the company	
Communication	Both parties have a dedicated person to coordinate communication	There is a dedicated person on both sides to coordinate communication	
	The company often meets with the farmer face to face to discuss matters related to the cooperation	I have regular face to face meetings with the company about the cooperation	
	Both parties are patient in resolving conflicts and misunderstandings when cooperation occurs	Both parties are patient in resolving conflicts and misunderstandings when they arise	
	Both sides often provide timely information to help each other	Information is often provided in a timely manner to help each other	
Supply Chain Transparency	Farmers share marketing information with us	The company shares sales information with me	[62,63]
	Demand forecasts are shared with us	The company shares demand forecasts with me	
	Farmers share inventory information with us	Companies share inventory information with me	
	Farmers share production planning information with us	The company shares production planning information with me	
Income Increase	Our sales revenue is increased by working with each other	We work together to increase my sales revenue	[35,64]
	We increase our supply capacity by working with each other	Working with each other increases my production capacity	
	Working with farmers provides a quick return on investment	Working with a company gives you a quick return on your investment	
	Stable source of profit by working with farmers	Working with a company provides a stable source of profit	

**Table 5:** Reliability analysis: companies and farmers.

Constructs	Companies			Farmers		
	Numbers of Items	Cronbach’s α	CR	Numbers of Items	Cronbach’s α	CR
Shared values	3	0.553	0.770	3	0.634	0.804
Reciprocity	4	0.785	0.862	4	0.722	0.822
Communication	4	0.867	0.910	4	0.806	0.873
Supply chain transparency	4	0.888	0.922	4	0.794	0.863
Income increase	4	0.829	0.887	4	0.765	0.850

**Table 6:** Convergent validity analysis: companies.

Constructs	Loadings	Standard Deviation	T Value	*p*	AVE
Shared values	0.839	0.036	23.495	***	0.536
	0.780	0.046	17.121	***	
	0.542	0.092	5.883	***	
Reciprocity	0.750	0.041	18.096	***	0.609
	0.801	0.037	19.856	***	
	0.801	0.035	22.777	***	
	0.829	0.027	30.766	***	
Communication	0.740	0.038	19.420	***	0.718
	0.885	0.017	52.578	***	
	0.867	0.018	46.995	***	
	0.888	0.016	55.826	***	
Supply chain transparency	0.866	0.023	38.493	***	0.748
	0.887	0.017	52.407	***	
	0.826	0.042	19.723	***	
	0.879	0.018	47.570	***	
Income increase	0.875	0.014	60.694	***	0.662
	0.764	0.028	27.496	***	
	0.830	0.026	31.870	***	
	0.779	0.034	22.991	***	

Note. *** *p* < 0.001.

**Table 7:** Convergent validity analysis: farmers.

Constructs	Loadings	Standard Deviation	T Value	*p*	AVE
Shared values	0.769	0.027	28.861	***	0.577
	0.780	0.036	21.758	***	
	0.729	0.035	21.061	***	
Reciprocity	0.797	0.027	29.904	***	0.537
	0.758	0.033	22.790	***	
	0.692	0.047	14.732	***	
	0.677	0.047	14.324	***	
Communication	0.781	0.032	24.255	***	0.633
	0.849	0.019	44.556	***	
	0.810	0.021	39.105	***	
	0.738	0.043	17.321	***	
Supply chain transparency	0.822	0.022	36.655	***	0.618
	0.862	0.020	43.874	***	
	0.867	0.018	48.846	***	
	0.550	0.053	10.408	***	
Income increase	0.776	0.024	32.235	***	0.587
	0.793	0.027	29.111	***	
	0.756	0.028	27.121	***	
	0.737	0.034	21.795	***	

Note. *** *p* < 0.001.

**Table 8:** Discriminant validity analysis: companies.

	Shared Values	Reciprocity	Communication	Supply Chain Transparency	Income Increase
Shared values	0.732				
Reciprocity	0.541	0.780			
Communication	0.621	0.702	0.847		
Supply chain transparency	0.540	0.582	0.654	0.865	
Income increase	0.539	0.693	0.649	0.537	0.813

Note. the value on the diagonal is the square root of the corresponding AVE value, and the value on the non-diagonal is the correlation coefficient between variables.

**Table 9:** Discriminant validity analysis: farmers.

	Shared Values	Reciprocity	Communication	Supply Chain Transparency	Income Increase
Shared values	0.760				
Reciprocity	0.634	0.733			
Communication	0.594	0.570	0.796		
Supply chain transparency	0.454	0.482	0.509	0.786	
Income increase	0.580	0.622	0.462	0.449	0.766

Note. the value on the diagonal is the square root of the corresponding AVE value, and the value on the non-diagonal is the correlation coefficient between variables.

**Table 10:** Moderating effects of supply chain transparency.

Performance: Income Increase						
Constructs	Company’s Perspective			Farmers’ Perspective		
	Model 1	Model 2	Model 3	Model 1	Model 2	Model 3
Shared values	0.173 **	0.160 *	0.207 *	0.317 ***	0.299 ***	0.295 ***
Communication	0.218 **	0.188 *	0.269 ***	0.083 ^ns^	0.037 ^ns^	0.044 ^ns^
Reciprocity	0.437 ***	0.421 ***	0.390 ***	0.342 ***	0.310 ***	0.274 ***
Supply chain transparency		0.075 ^ns^	0.048 ^ns^		0.148 ***	0.130 **
Shared values × supply chain transparency			0.030 ^ns^			−0.034 ^ns^
Communication × supply chain transparency			0.143 *			0.079 ^ns^
Reciprocity × supply chain transparency			0.028 ^ns^			−0.184 ***
R^2^	0.528	0.530	0.554	0.416	0.432	0.461
Adjusted R^2^	0.520	0.521	0.538	0.413	0.427	0.452
ΔR^2^	0.528 ***	0.003 ^ns^	0.024 *	0.416 ***	0.015 ***	0.029 ***
ΔF	73.313 ***	1.245 ^ns^	3.461 *	108.699 ***	12.320 ***	8.088 ***

Note. * *p* < 0.05, ** *p* < 0.01, *** *p* < 0.001, ns: not significant.

## Data Availability

The data presented in this study are available on request from the corresponding author.
